# Supplementary material for: Large language models-enabled digital twins for precision medicine in rare gynecological tumors
Source: NPJ Digit Med. 2025 Jul 9;8:420. doi: 10.1038/s41746-025-01810-z (PMC12241315; doi:10.1038/s41746-025-01810-z)
Supplement: Supplementary file 1 — Supplements. [file 41746_2025_1810_MOESM1_ESM.pdf]

Supplements

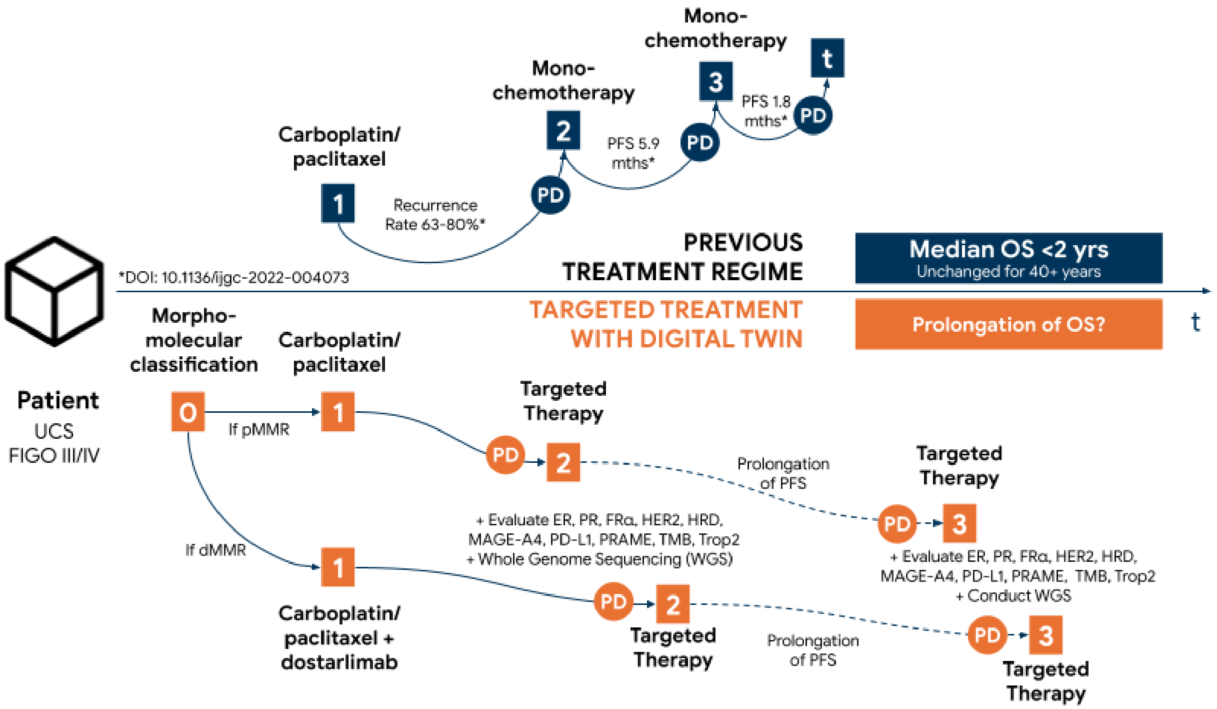

Supplementary Figure 1 The previous treatment regime did not mention the addition of dostarlimab as the consensus statement was published before EMA approval of dostarlimab on October 12, 2023. Reported response rates and median PFS with third-line therapy were 5.5% and 1.8 months, respectively. The 5-year overall survival rate has not changed in decades (31.9% in 1975 to 33.8% in 2012).[12] The targeted treatment approach could result in a prolongation of PFS and consequently a better OS.

Abbreviations: UCS – Uterine Carcinosarcoma. FIGO – International Federation of Gynecology and Obstetrics. PD – Progressive Disease. PFS – Progression-Free Survival. OS – Overall Survival. Yrs – years. pMMR – proficient Mismatch Repair. dMMR – deficient Mismatch Repair. ER – Estrogen Receptor. PR – Progesterone Receptor. FRα – Folate Receptor Alpha. HRD – Homologous Recombination Deficiency. MAGE-A4 – Melanoma-Associated Antigen A4. PD-L1 - Programmed Cell Death-Ligand 1. PRAME – Preferentially Expressed Antigen in Melanoma. TMB – Tumor Mutational Burden. Trop2 – Human trophoblastic cell surface antigen 2. WGS – Whole Genome Sequencing.

Supplementary Table 1 Detailed results of MTB for case 1

**Submitting hospital**	**Block material**	**Biomarkers tested before MTB**	**Tumor cell count**	**Panel used**	**Single Nucleotide Variants (SNV)/Indel**	**Copy number changes**	**Splice variations/translocations**
TUM University Hospital, Munich, Bavaria, Germany	Supracervical lymph node metastasis (2021)	ER: 80%<br>PR: 3%<br>HER2: Positive	60%	TSO 500 (DNA)/ TST 170 (RNA)	None detected	None detected	None detected
**Gene**	**Reference number**	**Exon**	**cDNA**	**Protein**	**Allele frequency**	**COSMIC database v90**	**Class**
TP53	NM_000546	4	c.150delT	p.I50Mfs*73	14%	COSV52758078	4
PPP2R1A	NM_014225	6	c.771G>T	p.W257C	12%	COSV59043009	4
NOTCH4	NM_004557	18	c.2780G>A	p.C927Y	8%	No entry	3
RUNX1	NM_001754	9	c.1070C>T	p.P357L	9%	No entry	3
AR	NM_000044	1	c.476C>G	p.A159G	7%	No entry	3

Abbreviations: MTB – Molecular Tumor Board. TUM – Technical University of Munich. ER – Estrogen Receptor. PR – Progesterone Receptor. TSO 500 - TruSight Oncology 500. TST 170 - TruSight Tumor 170.

Supplementary Table 2 Data structure for RGT Digital Twin

Attribute	Data Type	Description
n	Integer	Number of patients in the study
age	String	Age of patients in the study; string as many studies contain ranges
gender	String	Gender(s) of patients in the study
race	String	Race(s) of patients in the study
diagnosis	String	Diagnosis of patients in the study
biomarkers	{ 'pd-l1': String, 'tmb/mb': String, 'msi/mss':String, 'others': String }	Biomarkers (e.g., PD-L1) determined and discussed in the study; returned as dictionary for simplified analysis
previous treatments	String	Description of previous treatments (and response)
study treatment	String	Treatments discussed in the study
study treatment response	{ 'treatment response': String, 'adverse effects': String }	Response to treatments discussed in the study including adverse effects; returned as dictionary for simplified analysis
PFS	String	Progression-free survival (PFS) in months reported for study treatment; string as many studies contain ranges
OS	String	Overall survival (OS) in months reported for patient cases; string as many studies contain ranges

Abbreviations: RGT – Rare Gynecological Tumor. PFS – Progression-Free Survival. OS – Overall Survival. PD-L1 – Programmed Cell Death-Ligand 1.

Supplementary Table 3 Baseline characteristics of institutional MTB cases at TUM University Hospital

ID	Gender	Primary site	Age at diagnosis	Stage at diagnosis	Sites of metastases	Time of MTB	Site sequenced	Tumor cell count	PD-L1	TMB (Mut/Mb)	MMR
1	female	Uterine Carcinosarcoma (UCS)	66	FIGO IIIC2, pT3a, pN2 (3/67), R0	Cervical lymph node metastasis, pelvic recurrence, retroperitoneal lymph node metastases	2021	Cervical lymph node metastasis	60%	CPS: 41 TPS: 3% IC: 40%	6.3 (intermediate)	pMMR/ MSS (3.6%)
2	female	Cervical Squamous Cell Caricnoma (CESC)	30	FIGO IB1, pT1b1, pNX, G3, R0	Pelvic recurrence, liver metastasis	2020	Primary surgery	70%	CPS: 75 TPS: 70% IC: 5%	0 (low)	pMMR/ MSS (1.11%)
3	female	Cervical Squamous Cell Caricnoma (CESC)	31	FIGO IVB, cT2a, pN1 (12/94), cM1 (PER)	Peritoneal metastases	2020	Peritoneal metastasis	75%	CPS: 40 TPS: 40% IC: <1%	3.1 (low)	pMMR/ MSS (0%)
4	female	Cervical Squamous Cell Caricnoma (CESC)	79	FIGO IVa, cT2b2, cN1, cM0, G2	Lymph node metastases	2021	Primary tumor biopsy	80%	CPS: 81 TPS: 80% IC: 1%	11 (intermediate)	pMMR/ MSS (4.6%)
5	female	Cervical Adenocarcinoma (CEAD)	32	FIGO IVB, cT2b, cN1, cM1 (LYM)	Lymph node metastases	2021	Ileocecal resection	40%	CPS: 95 TPS: 90% IC: 5%	5.5 (intermediate)	pMMR/ MSS (3.28%)
6	female	Uterine Serous Carcinoma (USC)	55	FIGO IA1, pT1a, pNx, L0, V0, Pn0, R0	Peritoneal metastases, lymph node metastases	2024	Inguinal lymph node metastasis	30%	CPS: 40 TPS: 30% IC: 8%	13.4 (intermediate)	pMMR/ MSS (1.89%)
7	male	Undifferentiated Sarcomatoid Carcinoma of the Pancreas	59	pT3, pN1 (3/81), L1, V1, Pn1, R0	Lcooregional recurrence, liver metastases	2022	Liver metastasis	70%	CPS: 85 TPS: 80% IC: 4%	3.2 (low)	pMMR/ MSS (2.61%)

Abbreviations: MTB – Molecular Tumor Board. TUM – Technical University of Munich. PD-L1 – Programmed Cell Death-Ligand 1. TMB – Tumor Mutational Burden. MMR – Mismatch Repair. FIGO - International Federation of Gynecology and Obstetrics. CPS – Combined Positive

Score. TPS – Tumor Proportion Score. IC –Tumor-Infiltrating Immune Cells. pMMR – proficient Mismatch Repair. MSS – Microsatellite Stable.

Supplementary Table 4 Seven studies on ICI treatment in UCS lacked patient-level data necessary for individual digital twin creation

Trial	Recruitment period	Phase	Experimental group	Control group	Sample size	Treatment response	Median follow-up (months)	Median PFS (months) experimental vs control
RUBY trial [55]	2019-2021	III	Carboplatin/paclitaxel (CP) + dostarlimab x 6 cycles + maintenance with dostarlimab	CP + placebo x 6 cycles + maintenance with placebo	Overall: 494<br>UCS: n = 44<br><br>dMMR: 118 (UCS: 5)<br>pMMR: 376 (UCS: 39)	Not stratified for UCS<br><br>Overall: Hazard Ratio (HR) = 0.64 (0.51-0.80) p <0.001<br><br>dMMR: HR = 0.28 (0.16-0.50) p <0.001<br><br>pMMR: HR = 0.76 (0.59-0.98)	Not stratified for UCS<br><br>Overall: 25.4<br>dMMR: 24.8<br>pMMR NA	NA
DUO-E trial [56]	2020-2022	III	CP + durvalumab x 6 cycles + maintenance with durvalumab	CP + placebo x 6 cycles + maintenance with placebo	Overall 479<br>UCS: n = 61<br><br>dMMR 95<br>pMMR 384	Overall: HR = 0.71 (0.57-0.89) p = 0.003<br>dMMR: HR = 0.42 (0.22-0.80)<br>pMMR: HR = 0.77 (0.60-0.97)<br><br>Histology: other, including UCS (27/39)<br>HR = 0.76 (0.46-1.25), n.s.	Not stratified for UCS<br><br>Control 12.6<br>Experimental 15.4<br>dMMR 10.2<br>pMMR 12.8	Not stratified for UCS<br><br>Overall 10.2 vs 9.6<br>dMMR NR vs 7<br>pMMR 9.9 vs. 9.7
AtTEnd trial [57]	2018-2022	III	CP + atezolizumab x 6 cycles + maintenance with atezolizumab	CP + placebo x 6 cycles + maintenance with placebo	Overall: 549<br>UCS: n = 50<br><br>dMMR: 125<br><br>pMMR: 409	Overall: HR = 0.74 (0.61-0.91), p = 0.02<br>UCS: HR = 0.88 (0.45-1.73), n.s.<br><br><br>dMMR: HR = 0.36 (0.23-0.57), p = 0.0005<br>UCS: HR = 0.41 (0.03-6.62), n.s.<br><br>pMMR: HR = 0.92 (0.73-1.16), n.s.<br><br>UCS: not specified	Not stratified for UCS<br><br>Overall: 28.3<br>dMMR: 26.2<br>pMMR NA	Not stratified for UCS<br><br>Overall: 10.1 vs 8.9<br>dMMR NR vs. 6.9<br>pMMR 9.5 vs 9.2
Single-center, randomized,	N/a	II	Arm 1: Durvalumab	None	Overall: 82	Overall:	N/a	N/a

Supplementary Table 4 Seven studies on ICI treatment in UCS lacked patient-level data necessary for individual digital twin creation

Trial	Recruitment period	Phase	Experimental group	Control group	Sample size	Treatment response	Median follow-up (months)	Median PFS (months) experimental vs control
open-label, phase II trial [33]	Data cut-off: December 2021		Arm 2: Durvalumab + tremelimumab		UCS: 16<br>Arm 1: 6<br>Arm 2: 10	Arm 1 Overall Response Rate (ORR): 10.8%<br>Arm 2 ORR: 5.3%<br><br>UCS: ORR: 0%		
NCI-MATCH (EAY131) [58]	2016-2017	II	Nivolumab	None	Overall: 42<br><br>UCS: n = 4	Not stratified for UCS<br><br>Overall ORR: 36%	17.3	Not stratified for UCS<br><br>6-month PFS rate: 51.3%<br>12-month PFS rate: 46.2%<br>18-month PFS rate: 31.4%
Retrospective institutional analysis from The University of Texas MD Anderson Cancer Center [59]	2019-2020	Retrospective study of institutional data	Pembrolizumab + lenvatinib<br><br>Recommended dose of lenvatinib vs. reduced dose of lenvatinib	None	Overall: n = 61<br>Recommended dose: n = 14<br>Reduced dose: n = 47<br>UCS: n = 16<br>Recommended dose: n = 3<br>Reduced dose: n = 13	ORR:<br>Overall: 36.1%<br>UCS: 25% (3/12)<br><br>Clinical benefit rate (CBR):<br>Overall: 68.9%<br>UCS: 58.3% (7/12)	Not stratified for UCS<br><br>Overall:<br>Recommended dose: 3.2<br>Reduced dose: 5.5	Not stratified for UCS<br><br>Recommended dose: 8.6<br>Reduced dose: 9.4
Multicenter, randomized, phase II trial [34]	2018-2019	II	Cabozantinib + nivolumab	None	Arm A: 36<br>Arm B: 18<br><br>Arm C:<br>UCS: n = 10<br>pMMR: 100%	Arm C (UCS):<br>ORR: 10%<br>1 PR, 5 SD	Overall (Arm A, B, C): 15.9	Arm C (UCS):<br>Median SD duration: 3.2 (range 2.8-7.6)

Abbreviations: ICI – Immune Checkpoint Inhibitors. UCS – Uterine Carcinosarcoma. PFS – Progression-Free Survival. n/a – not available. dMMR – deficient Mismatch Repair. pMMR – proficient Mismatch Repair. PR – Partial Response. SD – Stable Disease.

References

33. Rubinstein, M. M. *et al.* Durvalumab with or without tremelimumab in patients with persistent or recurrent endometrial cancer or endometrial carcinosarcoma: A randomized open-label phase 2 study. *Gynecol. Oncol.* **169**, 64–69 (2023).

34. Lheureux, S. *et al.* Translational randomized phase II trial of cabozantinib in combination with nivolumab in advanced, recurrent, or metastatic endometrial cancer. *J Immunother Cancer* **10**, (2022).

55. Mirza Mansoor R. *et al.* Dostarlimab for Primary Advanced or Recurrent Endometrial Cancer. *N. Engl. J. Med.* **388**, 2145–2158 (2023).

56. Westin, S. N. *et al.* Durvalumab Plus Carboplatin/Paclitaxel Followed by Maintenance Durvalumab With or Without Olaparib as First-Line Treatment for Advanced Endometrial Cancer: The Phase III DUO-E Trial. *J. Clin. Oncol.* **42**, 283–299 (2024).

57. Colombo, N. *et al.* Atezolizumab and chemotherapy for advanced or recurrent endometrial cancer (AtTEnd): a randomised, double-blind, placebo-controlled, phase 3 trial. *Lancet Oncol.* (2024) doi:10.1016/S1470-2045(24)00334-6.

58. Azad, N. S. *et al.* Nivolumab Is Effective in Mismatch Repair-Deficient Noncolorectal Cancers: Results From Arm Z1D-A Subprotocol of the NCI-MATCH (EAY131) Study. *J. Clin. Oncol.* **38**, 214–222 (2020).

59. How, J. A. *et al.* Toxicity and efficacy of the combination of pembrolizumab with recommended or reduced starting doses of lenvatinib for treatment of recurrent endometrial cancer. *Gynecol. Oncol.* **162**, 24–31 (2021).
